# *In vivo* Spectroscopic Evaluation of the Intraperitoneal Cavity in Canines^†^

**DOI:** 10.1111/php.13226

**Published:** 2020-03-20

**Authors:** Gwendolyn M. Cramer, Julia Sandell Meo, Jarod C. Finlay, Timothy C. Zhu, Theresa M. Busch, Keith A. Cengel

**Affiliations:** 1 Department of Radiation Oncology, University of Pennsylvania, Philadelphia, PA, United States; 2 Department of Physics and Astronomy, University of Pennsylvania, Philadelphia, PA, United States

## Abstract

As part of a preclinical trial for the treatment of peritoneal carcinomatosis (PC) with photodynamic therapy (PDT), we have assessed changes in optical properties, tissue oxygenation and drug concentration as a result of benzoporphyrin derivative (BPD)-mediated PDT using diffuse reflectance and fluorescence measurements. PDT can effectively treat superficial disease spread, but treatment efficacy is influenced by physical properties of the treated tissue which can change over the treatment time. In this study, healthy canines were given BPD and irradiated with 690 nm light during a partial bowel resection, and spectroscopic and fluorescence measurements were made using an in-house built spectroscopic probe. Hemoglobin concentration, oxygenation and optical properties were determined to be highly heterogeneous between canines and at different anatomical locations within the same subject, so further development of PDT dosimetry systems will need to address this patient and location-specific dose optimization. Compared to other photosensitizers, we found no apparent BPD photobleaching after PDT.

## INTRODUCTION

Photodynamic therapy (PDT) is a cancer treatment modality dependent on light, oxygen and a photosensitive drug. The photosensitizers porfimer sodium and verteporfin, as well as the photosensitizing precursor 5-aminolevulinic acid, are approved by the United States Food and Drug Administration (FDA) for a growing number of conditions (^1^). As PDT methods are improved for optimal treatment of bulky tumors, explicit PDT dosimetry must be investigated to enhance treatment efficacy.

Most current PDT delivery protocols treat a subject with a predetermined light dose regardless of the subject’s specific optical properties and photosensitizer uptake. It has been found that optical properties, oxygenation levels, and drug concentration can vary greatly both between tissue sites and subjects, and these variables are also altered by the PDT process (^2–5^). Treatment response consequently varies among subjects, revealing a need for determining individual optical properties, which affect light distribution and photosensitizing drug uptake and in turn determine tissue damage. With this information, explicit PDT dosimetry can be performed to improve selective tissue damage and treatment response. The investigation of tissue optical properties, oxygenation and photosensitizer concentration is useful to guide efforts toward improving PDT efficacy.

As a part of a preclinical trial for the treatment of peritoneal carcinomatosis (PC) using BPD (6), the behavior of this photosensitizer was evaluated via spectroscopic measurements, as well as the optical properties and oxygenation of tissues undergoing PDT. PC has a low survival outcome and is difficult to treat with traditional radiation methods due to the number of vital organs that need to be spared in the peritoneal region. PDT is well suited as a treatment modality for PC, as it can cause diffuse cell damage on the surface of the peritoneal cavity while sparing the surrounding tissues at greater depths.

Previous PDT studies treating PC using the photosensitizer Photofrin (7,8) show PDT to be a promising treatment modality but also point to several obstacles in treatment efficacy. The first of these challenges is inconsistent photosensitizer uptake in tumor, as previously described in research on Photofrin uptake in peritoneal malignancies (9). Second, if the treatment light does not penetrate tissue deeply enough, then insufficient cell death will occur. For this reason, in part, benzoporphyrin derivative (BPD) is used in this study. BPD is activated at 690 nm, compared to 630 for Photofrin. This means treatment light will penetrate to a greater degree in tissue and, also due to BPD’s higher quantum yield (10), cause greater cell damage. The goals of this preclinical study in canines were to evaluate the toxicity of BPD-PDT to identify clinically tolerable doses for PC, as described in (6), and to utilize noninvasive spectroscopy measurements to compare optical and physiological properties of peritoneal tissues to inform future preclinical and clinical peritoneal PDT trials, as described below.

Optical methods of monitoring tissue properties during PDT include reflectance spectroscopy techniques that allow for study of light propagation through tissue and therefore hemodynamic properties of the tissue, as well as fluorescence spectroscopy techniques to resolve the concentration of photosensitizer from among biological chromophores like hemoglobin. The spectroscopic techniques discussed here provide the optical properties μ_a_ (absorption coefficient) and ${\mu_{s}}^{\prime }$ (reduced scattering coefficient) which can be used to determine the light dose distribution (11). The optical properties, oxygenation levels and drug concentration provide essential information for PDT dosimetry. The techniques used in this study are easily applied to an in vivo human environment. We aim to characterize the effects of PDT on optical properties, oxygenation and drug concentration with the goal of better understanding and characterizing explicit PDT dosimetry.

## MATERIALS AND METHODS

### Preclinical trial.

All procedures were performed in the University of Pennsylvania Laboratory Animal Resources (ULAR) facilities using a protocol approved by the University of Pennsylvania Institutional Animal Care and Use Committee. A total of 15 young adult (aged 8–14 months) mongrel hounds weighing between 18.7 and 25 kg (mean 20.8 kg) were used. They were mixed gender (five male and 10 female). Two dogs were from Marshall Farms (North Rose, NY) and 13 were from Covance Research Products (Denver, PA). The dogs were quarantined for three days. Basic blood work, drawn from the cephalic vein, was performed and was found to ensure the safety and health of the subjects. Animals were premedicated with I.V. buprenorphine, meloxicam and diazepam. General anesthesia was induced with I.V. ketamine or propofol and maintained with inhalational isoflurane. Heart rate, respirations, pulseoximetry and end-tidal carbon dioxide were monitored continuously. Blood pressure was monitored invasively via an arterial catheter. In two cases, a urinary catheter was placed. A dose of I.V. cefazolin was given prior to skin incision and redosed every six hours as required. The dogs were prepped and draped in a sterile environment, and sterile technique was maintained throughout the procedure. A midline incision was made from xiphoid to pubis, and the abdominal contents were explored. Incisional biopsies of several tissues were taken before and after PDT, analyses of which are described in (6).

### In vivo PDT.

Each dog was given either 0.125 mg kg^−1^ (one dog) or 0.25 mg kg^−1^ (the remaining 14 dogs) BPD four hours before light administration. The dogs were kept in darkened rooms before light administration, and all lighting in the operating room was covered by thin sheets of polyester filter (GamColor #480, GAM, Los Angeles, CA). The purpose of this filter is to reduce the light intensity at the peak absorption of BPD to avoid activation of BPD outside the treatment field. Light was administered using an 8W diode laser with a peak at 690 nm (B&W Tek, Inc.). The light administration procedure was similar to that described previously in (2). Briefly, the mesentery and small bowel were irradiated piecewise using a light field projected by a microlens. The cavity and the remaining organs were subsequently irradiated using a diffusing light wand, which was moved throughout the cavity to ensure uniform distribution of light. The total fluence delivered to the tissue surface was monitored by isotropic detectors placed on the surface of each section of the bowel and at several locations in the cavity: (1) the right upper quadrant lateral to the liver, near the border of the diaphragmatic attachments, (2) the left upper quadrant similarly near the border of the diaphragmatic attachments, (3) the left lower quadrant in the paracolic gutter, (4) the right lower quadrant similarly and (5) the pelvis anterior to the dome of the bladder.

### Measurement techniques.

The optical properties *μ*_a_ and ${\mu_{s}}^{\prime }$ were measured before and after light administration. A probe with two light sources (one for fluorescence and one for reflectance measurements) was built in-house with 10 detector fibers and a push button for clinician control (see Fig. 1). The two source fibers were illuminated by a tungsten lamp (Avalight, Avantes, Inc.) and 403 nm diode laser (Power Technology, Inc.) for reflectance and fluorescence measurements, respectively. A CCD-based spectrograph (InSpectrum, Roper Scientific, Princeton, NJ) was coupled to the detector fibers, recording a spectrum for each fiber. Measurements were taken with the probe in contact with the tissue surface. Spectroscopic measurements were obtained in the following order by the CCD: diffuse reflectance (white light on), background (no source on) and fluorescence (403 nm laser on). The cycling sequence was repeated six times for each measurement and later averaged to reduce noise. The data were corrected for the CCD offset by subtraction of an averaged dark frame and for spectral variation of the tungsten lamp by dividing by the spectra measured in an integrating sphere with spectrally uniform reflectance. Systematic investigation of measurement errors for optical properties determined with this probe suggested that measurement uncertainties can be minimized if care is taken during measurement acquisition (e.g., avoiding difference in probe pressure) and a full reflectance spectral fitting algorithm is used for analysis (12). This probe and measurement techniques are additionally described in our previous work on ALA-PDT in head and neck carcinomas (3).

### Reflectance analysis.

Once the reflectance was processed, it was fit to extract the optical properties, hemoglobin concentration, oxygenation and drug concentration. The reflectance data were fit twice with two different fitting algorithms. The radially resolved fitting algorithm uses a model developed by Finlay and Foster (13) based on the hybrid P3 approximation presented by Hull and Foster (14). To fit for *μ*_a_, we assume the absorption spectrum is a linear combination of the spectra of the absorbers present in the tissue:

(1) $$\mu_{a}(\lambda )=\sum_{i}c_{i}\varepsilon_{i}\left(\lambda_{i}\right)$$

where *c_i_* is the concentration of the *i*^th^ absorber and *ε_i_* is the spectrum of the *i*^th^ absorber. In this study, the absorbers accounted for were oxyhemoglobin and deoxyhemoglobin. To fit for ${\mu_{s}}^{\prime }$, we assume scattering follows this form:

(2) $$\mu_{s}^{\prime }=A{\left(\lambda /\lambda_{0}\right)}^{-b}$$

where *A* and *b* are free parameters (15) and *λ*_0_ is the treatment wavelength, 690 nm. The data are fitted from 685 to 695 nm (at an increment of 1 nm) with the resulting value being average of the optical properties at each wavelength in that range.

The total hemoglobin and oxygenation levels are determined by fitting the data with a spectrally resolved fitting algorithm that uses the Hill equation (16), which is a function of deoxy- and oxyhemoglobin. The data are fitted using the Hill curve, implementing the same nonlinear optimization function *fminsearch* in MATLAB used in the radial fitting. We fitted the reflectance spectra acquired at the first four source-detector distances, where signal noise was at a minimum, individually and then took the average of the resulting values.

### Fluorescence analysis.

To analyze the diffuse fluorescence spectroscopy data and obtain photosensitizer concentrations in each tissue, we used the fitting algorithm developed by Finlay et al. (17). A basis spectrum comprised of a linear combination of autofluorescence and BPD fluorescence was acquired to determine the spectrum of BPD *in vivo*:

(3) $$Fn(\lambda )=C_{BPD}\times F_{BPD}(\lambda )+C_{af}\times F_{af}(\lambda )$$

where *C*_af_ is the autofluorescence component and *C*_BPD_ is the BPD concentration determined from the basis BPD and autofluorescence spectra. The basis spectrum for BPD (*F*_BPD_) was measured using two nude mice injected with BPD. After being given a tail injection of 0.25 mg kg^−1^, the mice underwent a 30-min drug-light interval and were treated with a 1-cm-diameter laser-beam size (laser was placed 8 cm away from the treatment site) at a power of 58.9 mW. Measurements were made before and after light delivery (treatment time was 55 min and 33 s) and at 5-min intervals during brief interruptions in treatment. The basis autofluorescence spectra (*F*_af_) were taken from averaging fourteen measurements made using the probe described above on the normal healthy skin of a nude mouse (Mouse 1) before BPD injection. To remove the characteristic spectral peak caused by a diet where chlorophyll is present, a best-fit linear combination of Gaussian functions was subtracted from the pre-drug measurements to obtain a smooth, characteristic autofluorescence spectrum (Fig. 2). The averaged autofluorescence of Mouse 1 (see Fig. 2A) was then subtracted from its averaged measurements made at the treatment site after light delivery to obtain the BPD basis spectrum.

## RESULTS

### Reflectance

The averaged value over the wavelength range 685–695 nm for $\mu_{a}$, and ${\mu_{s}}^{\prime }$ for each subject and for each tissue type was used to make Figs 3 and 4. These figures show the variation in $\mu_{a}$ and ${\mu_{s}}^{\prime }$ before and after PDT for each tissue type. Based on data available in the literature (18,19), the overall range in $\mu_{a}$ and ${\mu_{s}}^{\prime }$ falls within the expected range. The same tissue types that display a higher median value for absorption also demonstrate a higher median value for reduced scattering. There does not appear to be a trend in changes before and after light administration, which is consistent with the findings of Solonenko et al. (19). The overall change in ${\mu_{s}}^{\prime }$, however, is greater in relative magnitude than those observed in $\mu_{a}$.

The effective attenuation coefficient, $\mu_{\text{eff}}$, which is calculated from $\mu_{a}$ and ${\mu_{s}}^{\prime }$:

(4) $$\mu_{eff}=\sqrt{3\times \mu_{a}\left(\mu_{a}+\mu_{s}^{\prime }\right)}$$

is plotted for each tissue type in Fig. 5. This shows that tissues with higher $\mu_{a}$ and ${\mu_{s}}^{\prime }$ also have a shorter light penetration depth.

Physiological responses of tissue to PDT can include changes in oxygenation and blood flow due to vascular destruction. Values for tissue oxygen saturation $\left({StO}_{2}\right)$ are presented in Fig. 6. The spleen and aorta have the highest oxygen levels, while the liver and kidney are less oxygenated, and other tissues fall between 75% and 100% ${StO}_{2}$. Additional fits based on the Hill equation, as described in the methods, were concentrations of hemoglobin for each type of peritoneal tissue (Fig. 7). Spleen measurements of total hemoglobin are not included in this figure because of extremely high calculated hemoglobin concentrations (mean ± SD of 4030±1424μM pre-PDT and 3235±1221μM post-PDT), which are not physiologically relevant (20). Other than the spleen, measurements of the aorta show the highest hemoglobin concentrations.

### Fluorescence

Figure 8 plots the overall range and distribution of BPD concentration between animals and across tissue type before and after light administration, divided by the corresponding autofluorescence. There does not appear to be a trend in BPD concentration as a result of light administration. The abdominal wall, bowel and kidney measurements demonstrate a smaller overall range in drug concentration between animals. The aorta, bladder, colon, gallbladder and stomach demonstrate a greater variance over animals in BPD concentration. We were unable to obtain fits for BPD concentration in the spleen, which did not display any interpretable fluorescence signal due to its extremely high blood concentration and high *μ_a_*.

## DISCUSSION

*In vivo* optical properties for this large range of peritoneal tissues, as summarized in Table 1, were previously unevaluated. Solonenko et al. had reported the optical properties for canine large bowels, kidneys and prostates (19). The median reported ${\mu_{s}}^{\prime }$ for bowel ($11.1\;{cm}^{-1}$) falls within the range of data observed here. The median ${\mu_{s}}^{\prime }$ for canine kidney ($21.56\;{cm}^{-1}$) at 690 nm reported by Solonenko et al. is somewhat higher than what we observed. Our difficulties in obtaining proper fits for BPD concentration and hemoglobin levels in the spleen are likely due to the presence of blood in the field interfering with spectroscopy probe measurements, indicated by high $\mu_{a}$ values for spleen. Additionally, we found physiologically reasonable measurements of hemoglobin and oxygenation in all other tissues, although there was significant heterogeneity among dogs and tissues, and there were no clear changes caused by BPD-PDT.

The options for determining photosensitizer levels are, essentially, either *in vivo* fluorescence spectroscopy measurements utilized in this manuscript or *ex vivo* fluorometry measurements of resected tissue samples. Compared to other photosensitizers such as porfimer sodium and ALA/PpIX that can exhibit significant photobleaching after PDT light delivery *in vivo* (17,21–23), we see no obvious decrease in *in vivo* BPD fluorescence after PDT (Fig. 7). Additional *ex vivo* fluorometry measurements of BPD concentration in various tissue types in these canines also showed limited evidence of photobleaching (6,24). While photosensitizers can photobleach at different rates due to their individual properties (25), we suspect that the drug-light interval used here still permits enough BPD perfusion from blood vessels into tissue to minimize photobleaching effects of PDT (6). Furthermore, our fluorescence spectroscopy measurements presented here indicate highest BPD levels in the abdominal wall (Fig. 7), while *ex vivo* tissues show highest levels in the small bowel (6). Since the amount of BPD in a bulk sample of tissue does not necessarily correlate with the PDT dose (in terms of singlet oxygen production) received, spectroscopy measurements that can be sampled throughout the treatment sites may produce more relevant BPD concentrations and can also be used for real-time explicit dosimetry.

Characterizing the range of optical properties, tissue oxygenation and photosensitizer uptake *in vivo* is particularly useful for existing explicit PDT dosimetry models such as the macroscopic singlet oxygen model developed by our group (1,26–33). This model integrates the interactions between light dose, oxygenation and photosensitizer concentration. *In vivo* measurement of tissue oxygenation in particular is useful for increasing the accuracy of the treatment effect that is predicted by this model because most current protocols do not account for variations in tissue oxygenation or drug concentration throughout the treatment area (34). Characterization of the optical properties, which we found to be heterogeneous both between subjects and within each subject, can also improve the accuracy of this model which depends in part on the absorption and scattering of the treated tissue. The tissue optical properties and photosensitizer concentration, along with photosensitizer-specific photochemical parameters, can all be combined to generate explicit singlet oxygen dosimetry (1,28–33).

Another light fluence model applicable to the cavity geometry of the intraperitoneal region is the intracavitary integrating sphere model developed by WM Star (35). This model predicts an overall increase in light dose due to backscattering by the cavity walls; this increase is characterized by the integrating sphere factor β which is dependent on the optical properties of the cavity wall. Star’s model assumes uniform absorption and scattering over the entire cavity wall, using the mean value of each to calculate β. Our results here show the optical properties of the intraperitoneal cavity wall to be highly heterogeneous. By characterizing this heterogeneity, this model can be expanded in future work to calculate β based on *in vivo* measurements. However, our recent comparison between scatter light fluence rate and variations of β based on measured optical properties in patients from a Photofrin-mediated pleural PDT clinical protocol indicates that resulting scattered light fluence rate per source power $\left(\phi_{s}/S\right)$ fell within a relatively narrow range (36).

## Figures and Tables

**Figure 1.**
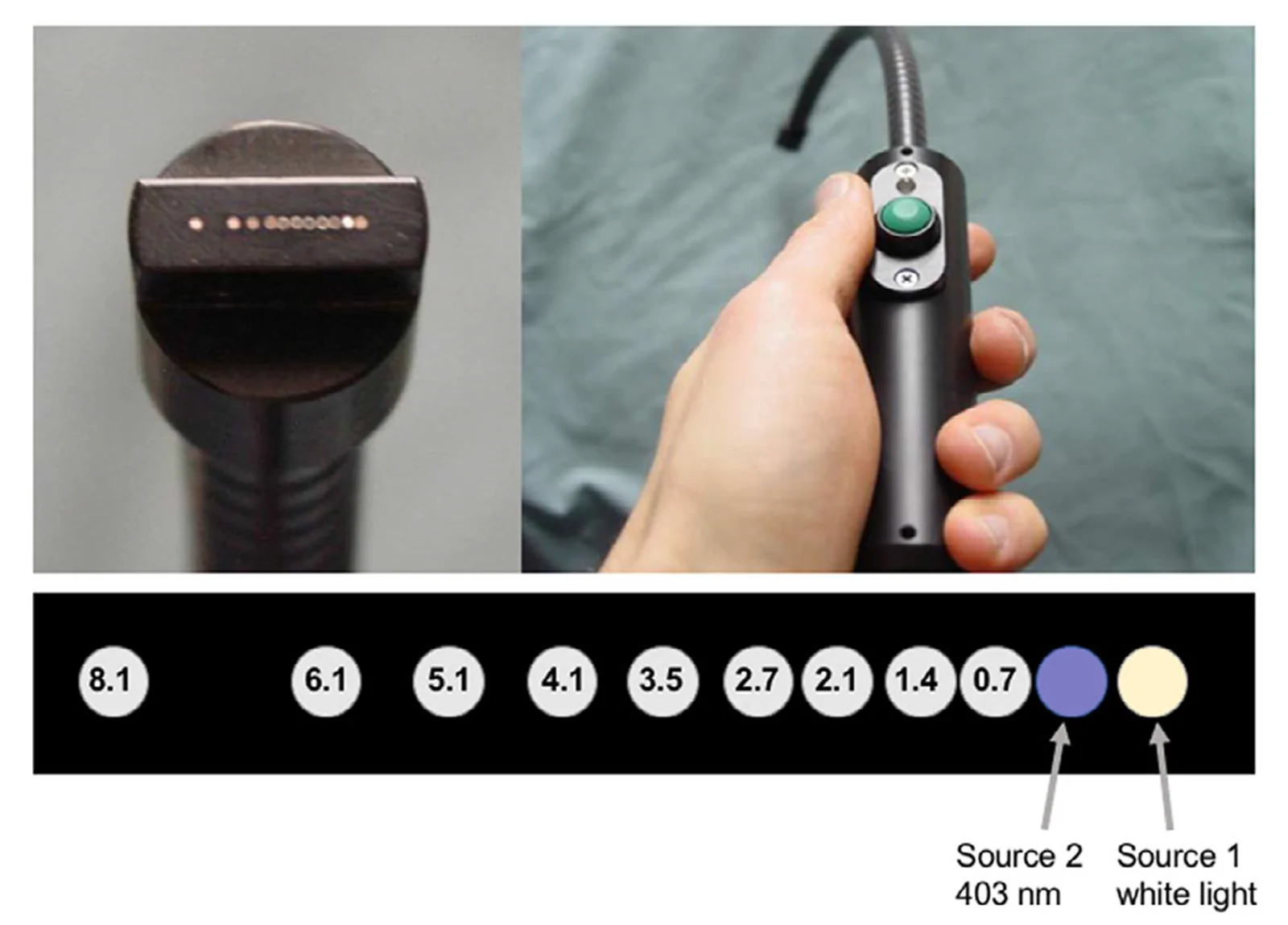
Probe used in clinic for spectroscopy measurements. The probe tip (left) displays the two light source fibers (one white light for reflectance measurements and a second 403 nm for fluorescence measurements) and the detectors at increasing distance from the light source. Distances from the 403 nm light source are shown in millimeters. The probe handle (right) is held by the clinician. To take a reading, the clinician pushes the green button once the light above the button indicates the instruments are ready for measurement.

**Figure 2.**
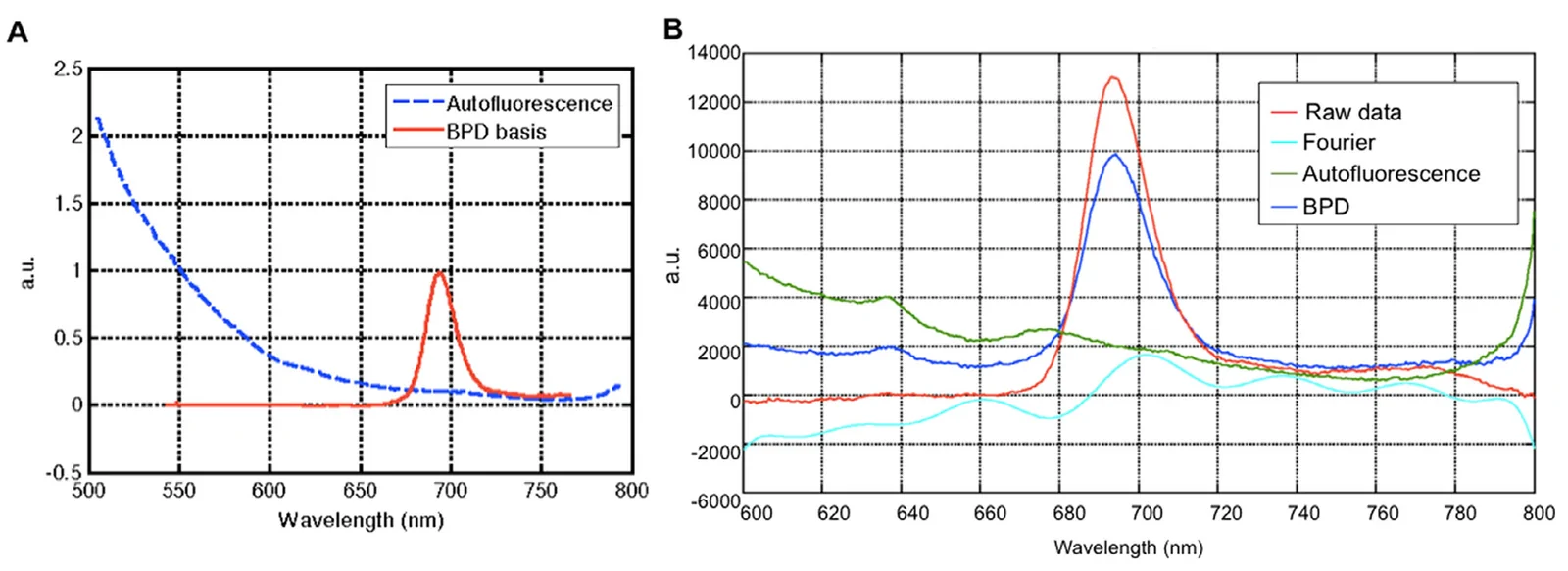
BPD fluorescence data fitting. (A) BPD basis spectrum (red) obtained from nude mice measurements and autofluorescence spectrum (blue). These spectra were used when fitting measured fluorescence data to determine drug concentration. (B) An example of fluorescence data fitting using the basis spectra in Fig. 2A including the Fourier components added to account for unknown fluorophores potentially affecting the measured spectrum.

**Figure 3.**
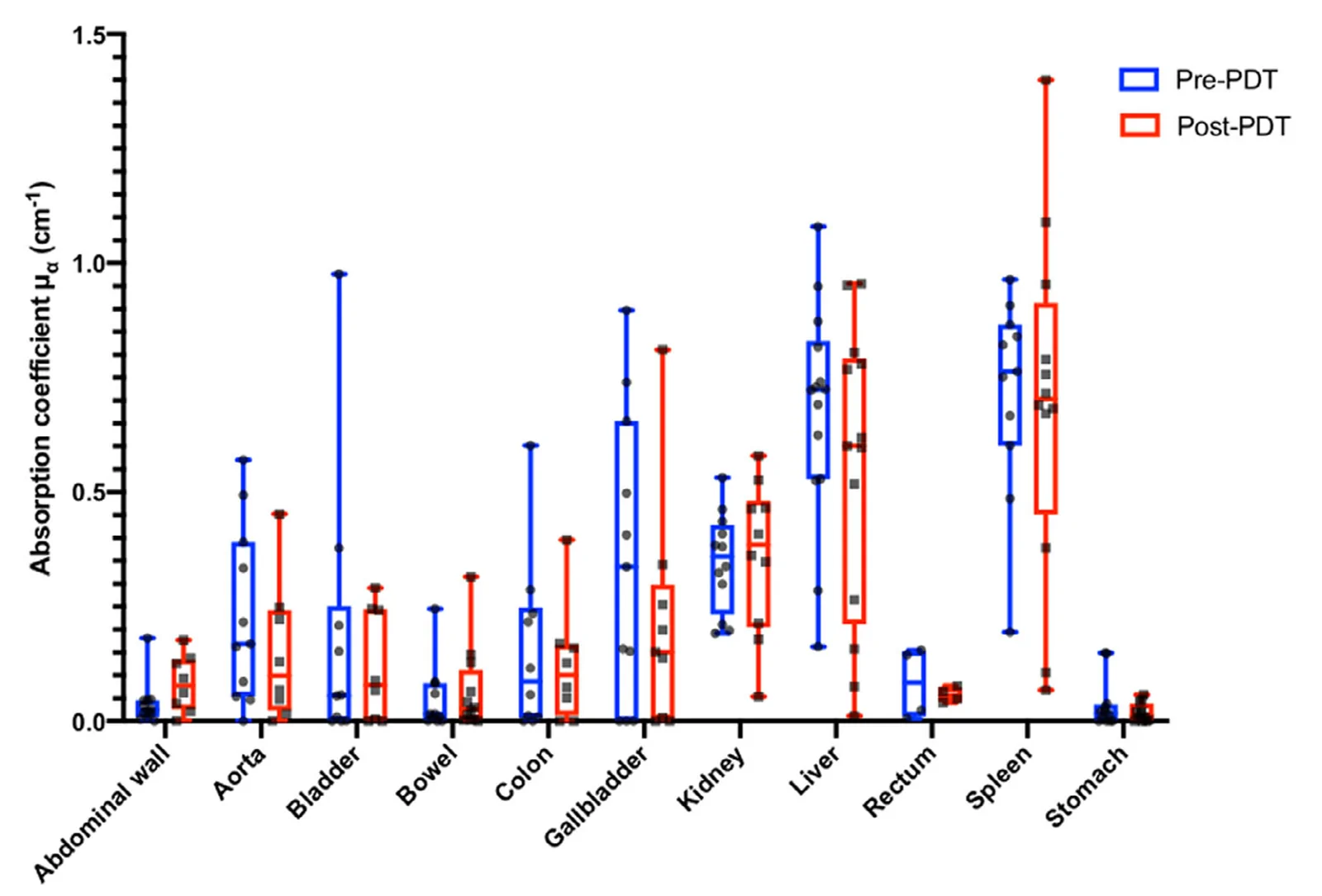
Absorption coefficient pre-PDT and post-PDT. Boxplots showing all *μ_a_* data points for pre-PDT (red) and post-PDT (blue) for each tissue type, with a line at the median. Note the median of *μ_a_* is higher for gallbladder, kidney, liver and spleen tissue compared to the other tissue types.

**Figure 4.**
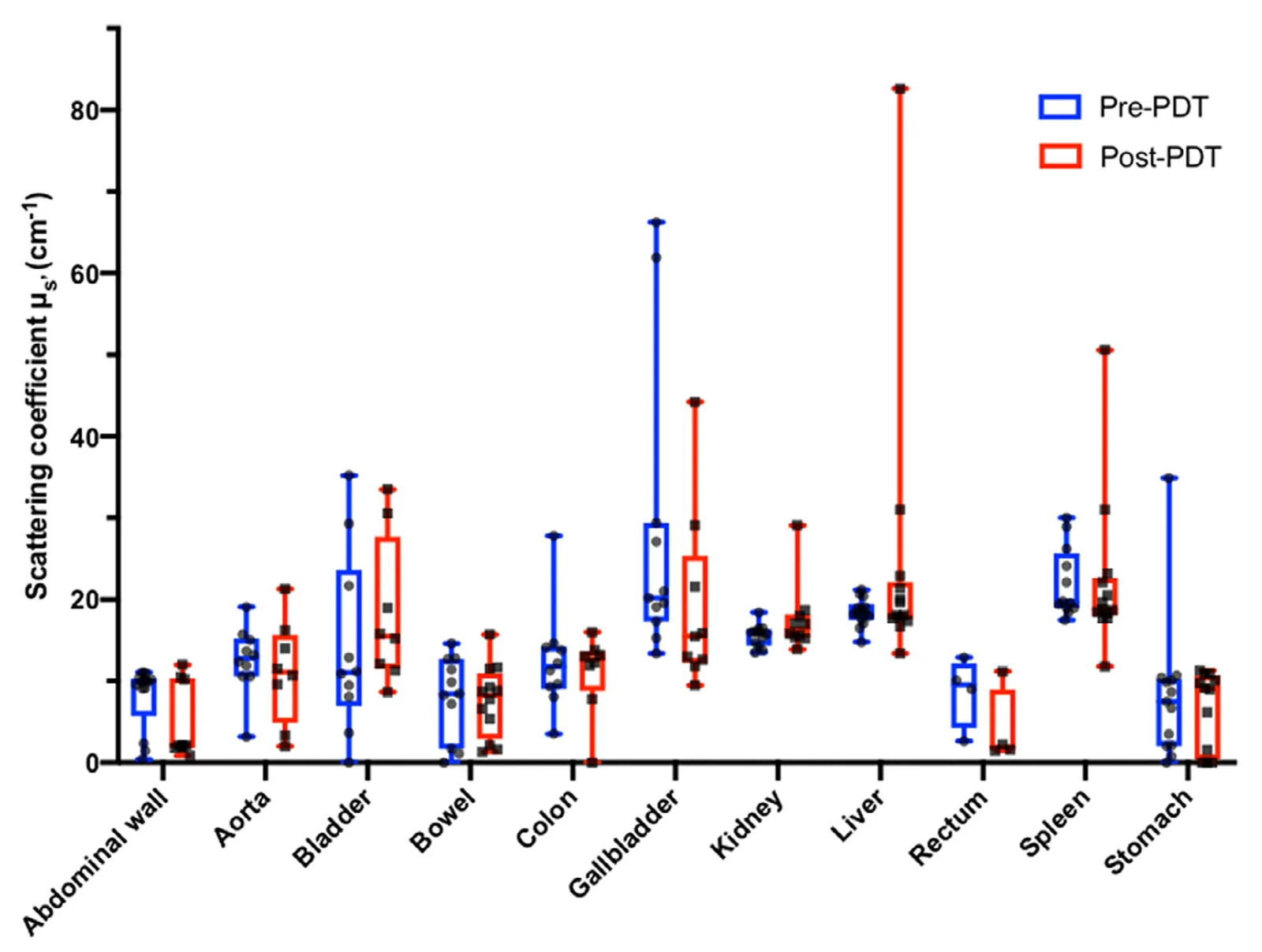
Reduced scattering coefficient pre-PDT and post-PDT. The pre-PDT (red) and post-PDT (blue) ${\mu_{s}}^{\prime }$ data are shown for each tissue type. The same tissues that demonstrate higher absorption also demonstrate higher reduced scattering.

**Figure 5.**
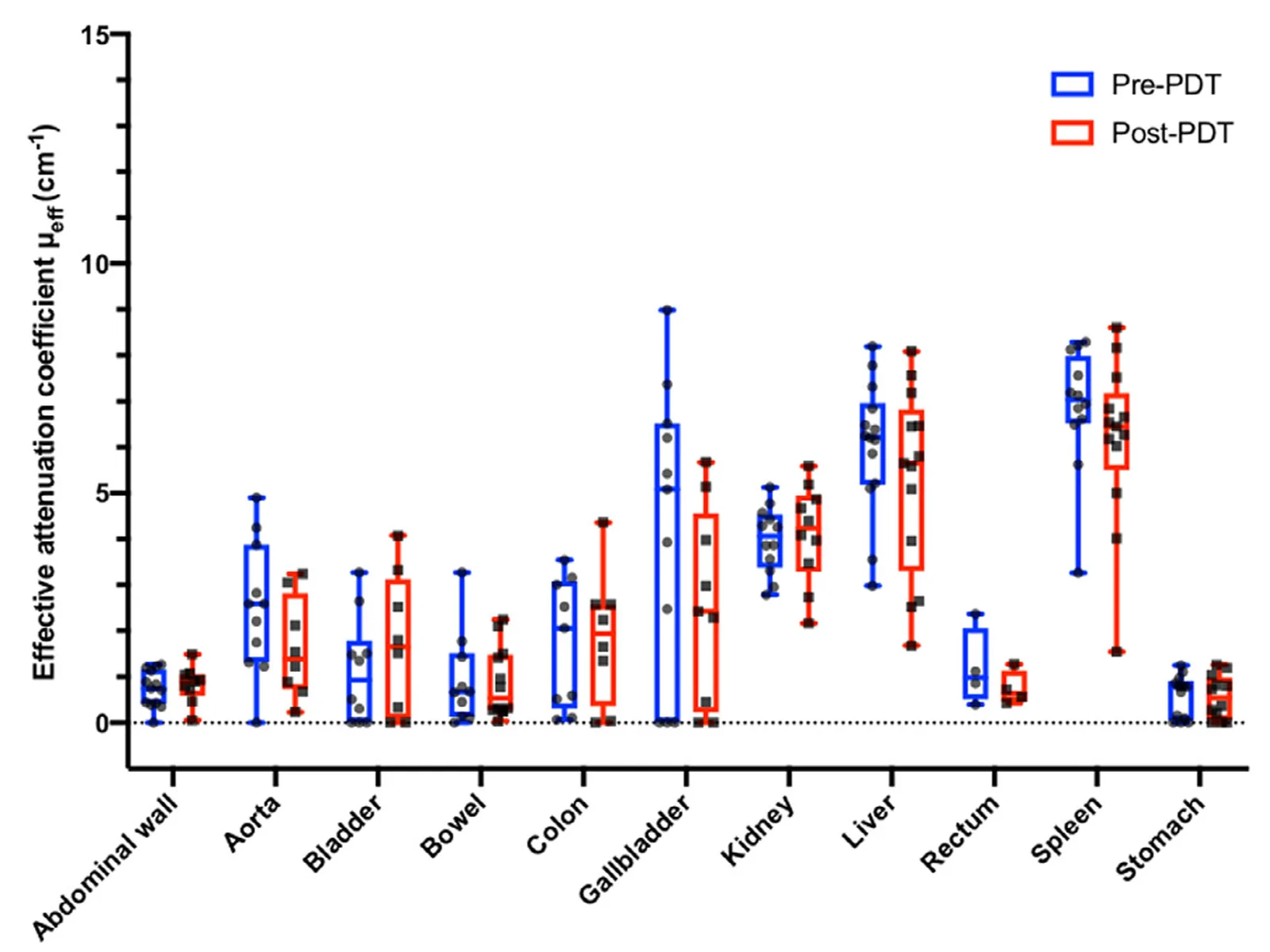
Effective attenuation coefficient pre-PDT and post-PDT. The pre-PDT (red) and post-PDT (blue) *μ*_eff_ data are shown for each tissue type. Tissues with higher *μ_a_* and *μ_s_* also have higher *μ*_eff_, with the most variability in gallbladder tissue.

**Figure 6.**
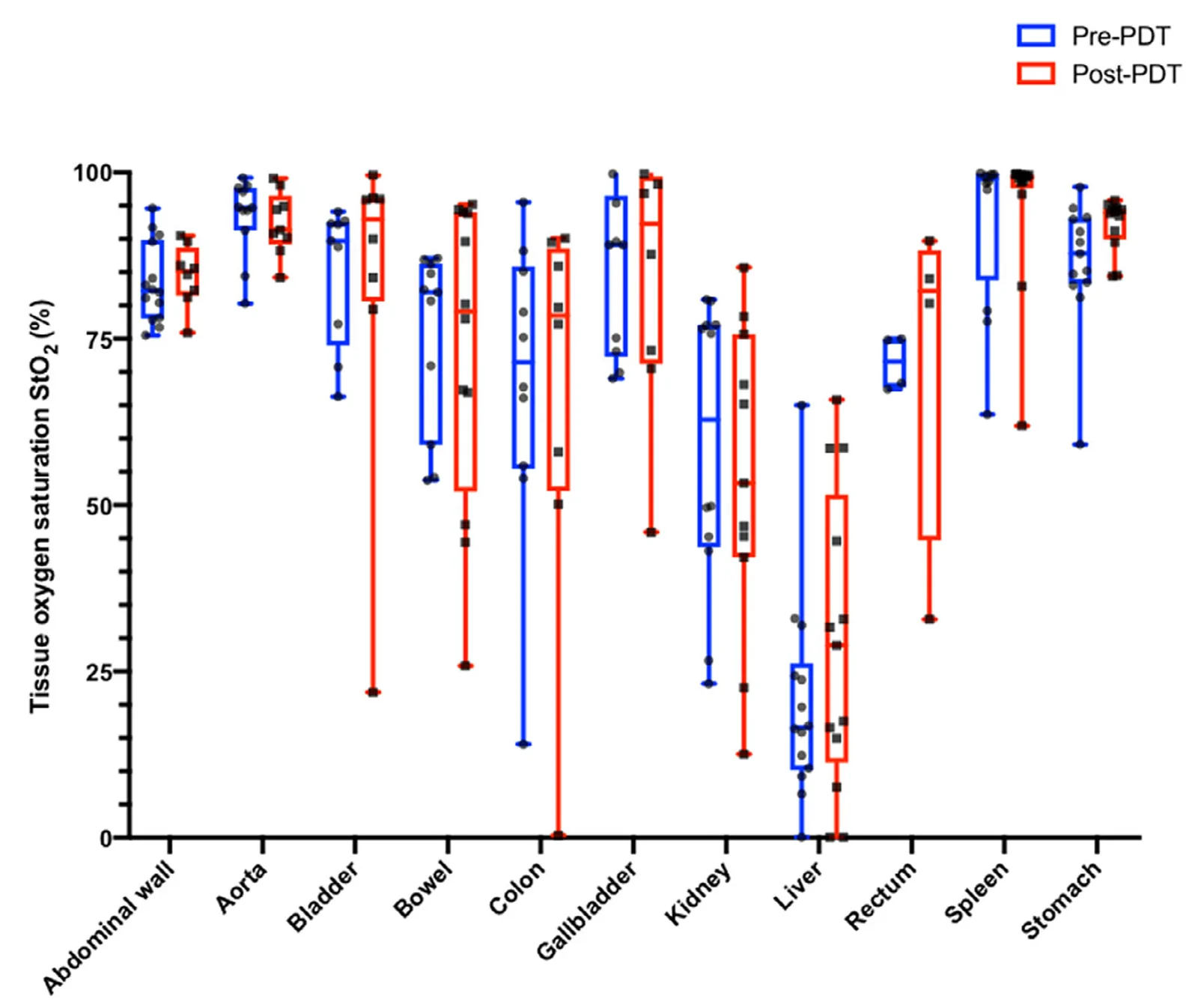
Hemoglobin pre-PDT and post-PDT. The total hemoglobin concentration (in μM) within pre-PDT and post-PDT measurements obtained using spectral fitting described in the methods. Spleen measurements are not included in this figure because of extremely high calculated hemoglobin concentrations (with means ± SD of 4030 ± 1424 pre-PDT and 3235 ± 1221 post-PDT)

**Figure 7.**
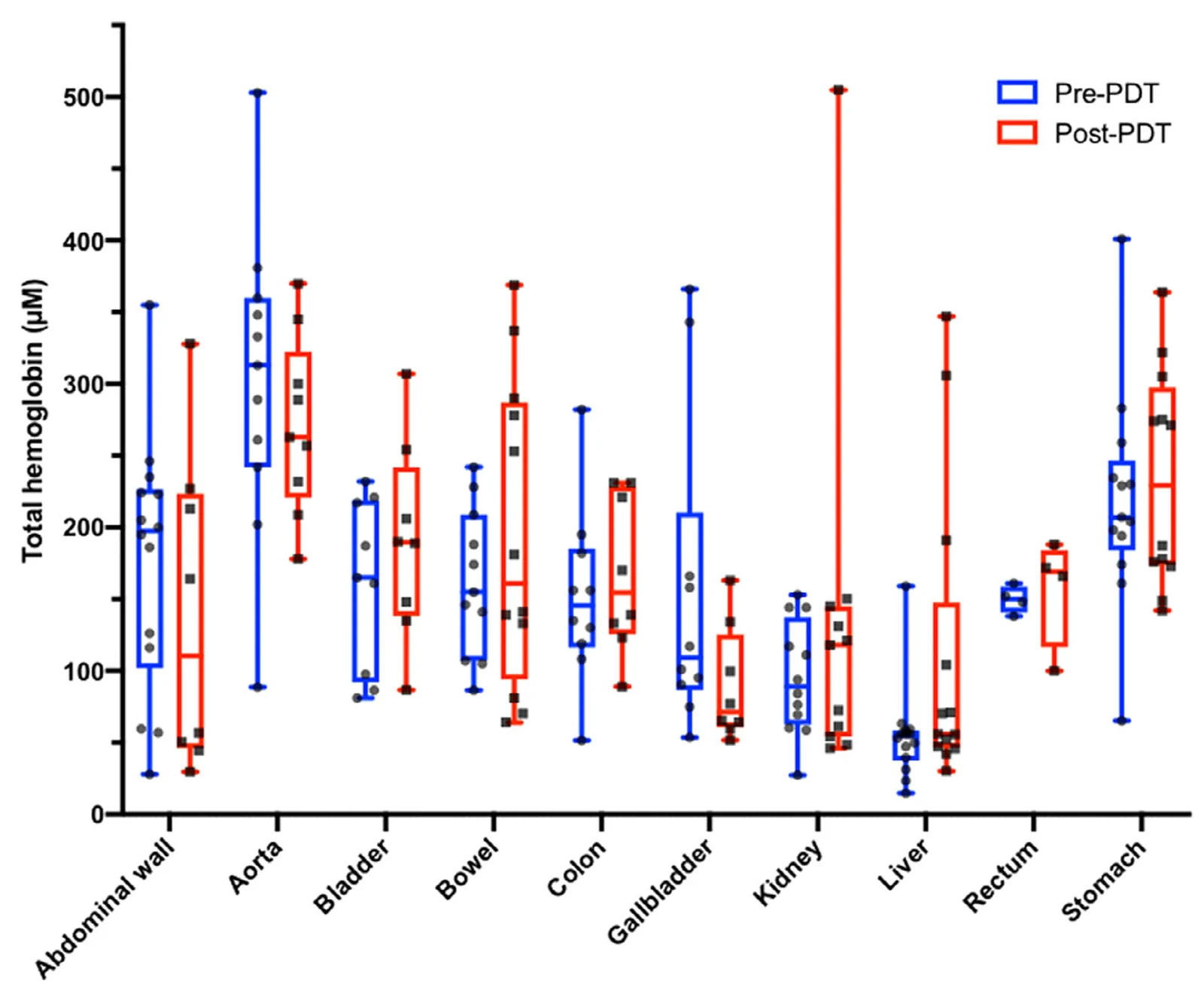
BPD-PDT does not change normal tissue oxygenation. StO_2_ percentages are plotted for each tissue type before (blue) and after (red) PDT. The aorta and spleen have the highest oxygenation of all the tissues evaluated.

**Figure 8.**
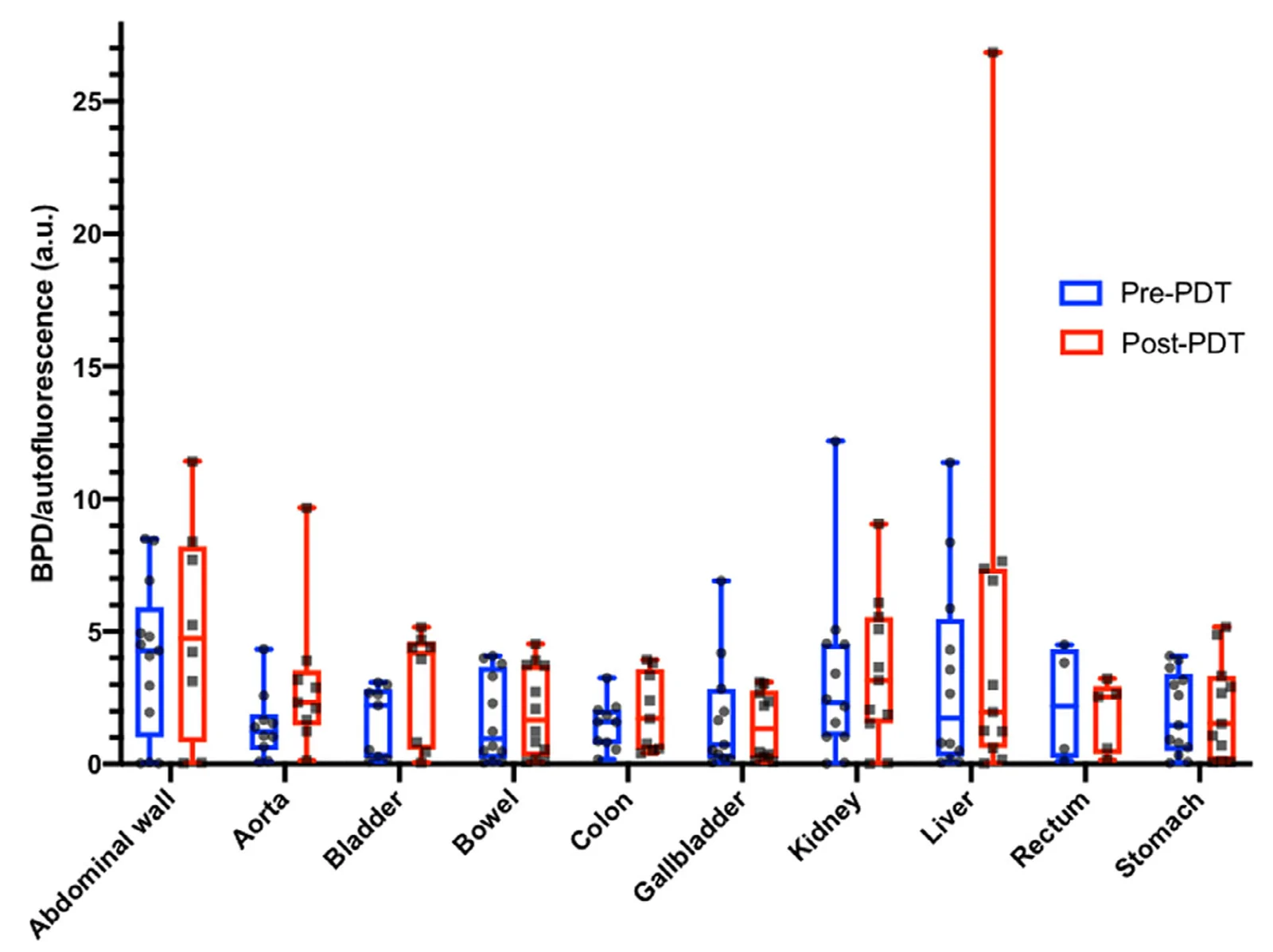
Light exposure does not photobleach BPD in healthy peritoneal tissues. BPD concentration measurements in peritoneal tissues were normalized by dividing by individual sample autofluorescence. Spleen measurements are not included here because the high μ_a_ prevented reasonable spectral fitting. The dog that received 0.125 mg kg^−1^ BPD has tissue photosensitizer measurements within the range of all other animals receiving 0.25 mg kg^−1^ BPD.

**Table 1.** Summary of optical properties before PDT, showing means (standard deviation) for each tissue type.

Tissue	*μ*_a_ (cm^−1^)	${\mu_{s}}^{\prime }$ (cm^−1^)	*μ*_eff_ (cm^−1^)	Hemoglobin (μM)	StO_2_ (%)
Abdominal wall	0.04 (0.05)	8.05 (3.86)	0.74 (0.40)	175.4 (88.9)	83.4 (6.0)
Aorta	0.23 (0.19)	12.54 (4.17)	2.50 (1.44)	301.9 (106.9)	93.3 (5.9)
Bladder	0.18 (0.30)	14.24 (11.15)	1.11 (1.16)	160.9 (59.7)	84.9 (10.6)
Bowel	0.05 (0.07)	8.01 (5.06)	0.93 (1.00)	161.9 (51.5)	75.3 (13.5)
Colon	0.15 (0.19)	12.44 (6.34)	1.73 (1.41)	151.4 (61.1)	68.1 (23.3)
Gallbladder	0.36 (0.32)	28.23 (18.33)	4.18 (3.17)	156.5 (110.0)	85.0 (12.2)
Kidney	0.35 (0.11)	15.58 (1.36)	3.98 (0.73)	94.9 (39.5)	58.8 (21.6)
Liver	0.67 (0.24)	18.43 (1.69)	6.02 (1.45)	54.7 (33.3)	20.3 (15.9)
Rectum	0.08 (0.08)	8.67 (4.33)	1.18 (0.85)	149.8 (9.5)	71.4 (4.1)
Spleen	0.72 (0.22)	22.00 (4.29)	6.86 (1.38)	4030.0 (1424.2)*	92.8 (12.2)
Stomach	0.04 (0.05)	8.25 (8.91)	0.57 (0.46)	218.4 (76.3)	86.4 (9.6)

* Spleen hemoglobin calculations are outside of a physiologically reasonable range.
